# The effect of desire to be liked and social appearance anxiety on aesthetic surgery acceptance in female nurses

**DOI:** 10.1186/s12912-024-02147-w

**Published:** 2024-07-08

**Authors:** Yaşar Demir, Erhan Dağ, Pınar Karakuş, Zeynep Aydın Kılınç

**Affiliations:** 1 Department of Statistics, Samsun Training and Researh Hospital, Samsun, Türkiye; 2 Gediz Health Services Vocational School, Kütahya Health Sciences University, Kütahya, Türkiye; 3 Graduate School of Education, Alanya Alaaddin Keykubat University, Alanya, Türkiye; 4 Alanya District Directorate of Health, Alanya/Antalya, Türkiye

**Keywords:** Social appearance anxiety, Desire to be liked, Aesthetic surgery Acceptance, Women, Nurses

## Abstract

**Background:**

A rapid increase has been observed in aesthetic surgery procedures in recent years and it has been determined that females have more aesthetic procedures. While different groups of female groups were taken as a sample in the studies, no study on female nurses was found. In this study, it is thought that psychological reasons such as stress, anxiety, desire to be liked and lack of self-confidence that lead women to plastic surgery will reduce the professional performance of nurses and this situation will create additional workload for other colleagues. Therefore, the aim of the study is to reveal the effect of desire to be liked and social appearance anxiety on the acceptance of female nurses to plastic surgery.

**Methods:**

The population of the cross-sectional study consisted of 243.565 nurses working in public, private and university hospitals in Turkey. A questionnaire form was used as a data collection tool in the study. In the first part of the form, there are statements aiming to reveal the socio-demographic characteristics, social media usage levels and aesthetic surgery experiences of nurses, and in the second part, there is a desire to be liked scale, social appearance anxiety scale and aesthetic surgery acceptance scale. The 1004 questionnaire forms collected as a result of the study were subjected to percentage, frequency, correlation and regression analysis.

**Results:**

41.2% of the nurses have had aesthetic procedures before and 68.4% of them want to have aesthetic procedures when there is an area they do not like in their body. A strong positive relationship was found between the desire to be liked, social appearance anxiety, and aesthetic surgery acceptance (*r* > 0.500, *p* < 0.01). Aesthetic surgery acceptance is affected by the desire to be liked and by social appearance anxiety.

**Conclusion:**

In the study, it was determined that social appearance anxiety and the desire to be liked led female nurses to plastic surgery. According to the results of similar studies conducted in different groups, it may be recommended that awareness training be organized both in schools and through digital media about the consequences of unnecessary plastic surgery.

## Introduction

Many individuals worldwide resort to aesthetic surgery for various reasons, ranging from correction of birth-related anomalies to correction of appearance disorders due to aging [1]. The number of aesthetic surgeries performed between 2018 and 2022 increased by 43.1% to 14.986.982, and the number of nonsurgical aesthetic procedures increased by 57.8% to 18.857.311. In 2022, the most common aesthetic surgery performed was liposuction (15.4%), while the most common nonsurgical aesthetic procedure was botulinum toxin (botox) (48.9%) [2, 3]. According to the International Society of Aesthetic Plastic Surgery (ISAPS) 2022 data, the five countries where both surgical and nonsurgical aesthetic procedures are performed the most are the United States, Brazil, Japan, Mexico, and Türkiye. According to the same data, the countries most likely to have both surgical and nonsurgical aesthetic procedures at a more affordable price are Mexico, Colombia, Thailand, Türkiye, and Spain [3].

Many studies in the relevant literature have examined the factors that direct individuals to plastic surgery [4–7]. According to the results of these studies, the most important factors that direct individuals to plastic surgery are social media and psychosocial factors [8–10]. Among psychosocial factors, the most important are social appearance anxiety, body dissatisfaction, and the desire to be liked when supported by social media [11–16].

The desire to be liked stems from individuals’ desire to be loved, respected, accepted, and satisfied with a sense of competence. This desire causes people to see themselves more positively and often tends to present themselves in a better light than they actually are. Therefore, this is commonly linked to the desire to be liked or admired [17–19]. The third level in Maslow’s hierarchy of needs is the need to be liked and to belong. Individuals’ desire to belong, to be accepted, and to be liked is analyzed in this step. According to this theory, since being liked is a strong need for individuals, not being liked and accepted by others negatively affects mental health, social harmony, academic success and life satisfaction [20]. Therefore, academic success, mental health and life satisfaction are negatively affected by social appearance anxiety as well as the desire to be liked [21].

Social appearance anxiety is a state of anxiety that occurs as a result of having negative thoughts about one’s own body and appearance and evaluating body image negatively. An individual’s anxiety about their appearance may cause them to feel worthless and lonely [21–23]. These individuals often have negative beliefs about themselves. However, this situation is not continuous. When individuals do not see any threat to themselves from others or when they are on their own, they can often have positive thoughts about their bodies. Moreover, this state of anxiety may vary according to the period and culture [24–27]. Especially in the last decade, with the increase in the use of social media due to technological developments, studies have shown that there has been an increase in individuals’ negative thoughts on body image [17, 27, 28]. Thus, individuals have shown an orientation toward plastic surgery both with the guidance of social media and the rapid development of health services. Similarly, many individuals turn to plastic surgery to be liked, accepted, appreciated, and admired by others to realize their desire to be liked and to correct their negative body image perception [18, 29–31].

Social appearance anxiety and the desire to be liked are related to negative body image [17, 21]. Body image perception is the most important component of self-esteem, especially in women [32]. Some studies have shown that the difference between current body image and ideal body image leads to dissatisfaction. Therefore, this dissatisfaction can cause eating behavior disorders, self-isolation, depression, loss of self-esteem and loss of well-being [33–35]. Therefore, these two psychosocial factors can be problematic in occupational groups working with labor-intensive workers, such as those in the nursing profession. The majority of those working in health services worldwide are female nurses.

Nursing is a professional profession that provides holistic care to individuals, families and society through the use of scientific knowledge and skills [36, 37]. The International Council of Nursing (ICN) defines nursing as “nursing is a professional group that protects and improves the health of the individual, family and society and participates in the recovery and rehabilitation process in case of illness. The nurse also develops the therapeutic and educational plans of the health team and participates in the implementation of these plans. “ [38].

Nurses aim to increase the health of individuals, families and societies; prevent disease; and restore health through the care they provide [36]. Therefore, the nurse who will fulfill such a task must be in a state of complete psychosocial and mental well-being.

Psychosocial factors that negatively affect individuals’ body dissatisfaction and self-image, such as social appearance anxiety and the desire to be liked, also negatively affect individuals’ level of mental well-being [39]. In addition, these factors lead to a decrease in the quality of life of individuals [40] and an increase in burnout levels [41]. Nurses who already have low life satisfaction and high burnout levels due to excessive workloads experience more stress and anxiety due to these factors [41]. As a result of anxiety and stress, nurses’ professional careers are damaged, organizational commitment levels decrease, turnover intentions increase and the quality of care they provide decreases [42].

The nursing profession involves professional groups in which close contact and communication with patients, their relatives and other employees are intensive, require a high level of attention and require a high level of well-being. In addition, nurses are the most important building blocks of health services. For this reason, the high psychosocial well-being of nurses is very important for the development and progress of individuals’ and society’s health. In addition, nurses have sufficient knowledge about all the physiological and pathological effects of surgical and nonsurgical procedures on the human body due to the education they receive. For these reasons, the aim of this study was to reveal the effect of the desire to be liked and social appearance anxiety on the acceptance of aesthetic surgery from the perspective of nurses. In the literature review, no study was found in which the desire to be liked, social appearance anxiety and aesthetic surgery acceptance were examined together in a sample of nurses. This situation reveals the originality of the study.

## Materials and methods

### Study design

The study is cross-sectional. The population of the study consisted of 243,565 nurses working in public, university, and private health institutions in Türkiye, according to the data of the Ministry of Health (2022) [43]. The minimum sample size of the study was determined to be 661, with a 99% confidence interval and 1% margin of error. Research data were collected between March 2024 and April 2024. Online surveys were used to collect the data. To obtain the target sample, the nonprobability sampling technique of convenience sampling was used [44]. In the explanation of the questionnaire, information about the purpose, possible risks and benefits of the study was provided. The first question of the questionnaire was “Do you agree to participate in this study?”. The questionnaire forms of the participants who answered “yes” to this question were included in the evaluation. To prevent the same participant from completing the questionnaire more than once, the block feature from the same IP and the same e-mail address was used. The survey was administered online and shared on different social media platforms, including Instagram, Facebook, and WhatsApp. Women nurses who had been working in hospitals within the scope of the study for at least one year and who had volunteered were included in the study. Employees who completed the questionnaire incompletely, had less than one year of employment and did not give voluntary consent were excluded. A total of 1004 surveys were collected.

### Data collection tool

A questionnaire consisting of 2 sections was used to collect the research data. In the first part of the questionnaire, 15 statements aimed to reveal the sociodemographic information, social media use and aesthetic surgery experiences of the nurses. The second part of the survey used the desire to be liked scale, social appearance anxiety scale and aesthetic surgery acceptance scale. For the use of the scales in the study, permission was obtained via e-mail from the academicians who conducted the Turkish validity and reliability study.

#### Desire to be liked scale

This scale was developed by Kaşıkara and Doğan (2017).

The scale comprises nine items and utilizes a 4-point Likert-type scoring system ranging from “1- Strongly Disagree” to “4- Strongly Agree”. The lowest possible score is obtained from the scale [9], and the highest possible score is 36. The higher the score obtained from the scale is, the greater the desire to be liked. The Cronbach’s alpha value of the scale was 0.82 [17]. In this study, the Cronbach’s alpha value of the scale was 0.847.

#### Social appearance anxiety scale

The scale was developed by Hart et al. (2008). The validity and reliability of the scale were assessed by Doğan (2010). It is a self-report scale with 16 statements developed to measure an individual’s emotional, cognitive, and behavioral concerns about his/her appearance; it uses a 5-point Likert-type scale [1] not at all appropriate and [5] completely appropriate. The 1st item of the scale is coded backward. The lowest possible score obtained from the SAAS, which measures social appearance anxiety in a single dimension, is 16, and the highest possible score is 80. High scores on the scale indicate that appearance anxiety is high. The Cronbach’s alpha value of the scale is 0.88 [21, 22]. In this study, the Cronbach’s alpha value of the scale was 0.93.

#### Aesthetic surgery Acceptance Scale

This scale was developed by Henderson-King and Henderson-King (2005), and a validity and reliability study of the Turkish translation of the scale in Türkiye was conducted by Karaca et al. (2017). The scale consists of 15 items and 3 subdimensions and is a 7-point Likert-type scale with 1 = Strongly disagree and 7 = Strongly agree. The subdimensions of the scale are personal, social and thoughts. The subdimensions of the scale are personal (items 1, 2, 4, 5 and 14), social (items 9, 11, 12, 13 and 15) and thoughts (items 3, 6, 7, 8 and 10). Only the 10th item contains a negative expression and is reverse coded, and an evaluation is made according to the three subdimensions and the total score of the scale. The score range of the ECBS is 15–105. An increase in the number of subdimensions and total scale score indicates positive attitudes toward aesthetic surgery. High-scale scores indicate that the person accepts plastic surgery. The Cronbach’s alpha of the scale is 0.91. [45, 46]. In this study, the Cronbach’s alpha value of the scale was 0.89.

### Statistics

We used the SPSS 26.00 program for data analysis. The skewness and kurtosis values of the data were examined first. Since the skewness and kurtosis values were found to be between + 1.5 and − 1.5 [47], it was assumed that the data were normally distributed. Pearson correlation analysis was performed to evaluate the associations between variables. Multiple regression analysis was used to evaluate the associations between variables.

## Results

Among the nurses who participated in the study, 51.29% were aged 30–39 years. The mean age of the nurses was 34.72 ± 6.97 years, and 65.11% were in the Y generation (born between 1980 and 1994). A total of 59.06% of the nurses were undergraduate graduates, 68.03% were married, and 84.76% worked both day and night (Table 1).

**Table 1:** Socio-demographic characteristics of participants

Variables	*N* (1004)	%
**Age**		
20–29	287	28,6
30–39	515	51,3
40–49	199	19,8
50–54	3	0,3
**Education**		
Associate degree	282	28,1
Bachelor’s degree	593	59,1
Master’s degree	124	12,4
PhD graduate	5	0,5
**Work year**		
1–5 year	164	16,3
6–10 year	298	29,7
11–15 year	377	37,5
16 +	165	16,4
**Marital status**		
Single	321	32,0
Married	683	68,0
**How to work**		
Daytime	71	7,1
Night	82	8,2
Both night and day	851	84,8
**Working Unit**		
Administrative Units	131	13,0
Health Care Services	873	87,0

All of the nurses had social media accounts. A total of 71.19% of them used Instagram the most. A total of 56.51% of the participants spent an average of 2–3 h daily on social media (Table 2).

**Table 2:** Participants’ social Media Use

Variables	*N* (1004)	%
**Using Social Media**		
Yes	1004	100,0
**Most used social media platform**		
Faceebook	212	21,1
İnstagram	715	71,2
Twitter X	36	3,6
Threads	4	0,4
Snapchat	15	1,5
Tik-Tok	22	2,2
**Average daily time spent on social media**		
0–1 h	362	36,1
2–3 h	568	56,6
4 h+	74	7,4
**Most shared post on social media**		
Photograph, text, comment, etc.	243	24,2
Story	658	65,5
Reels	103	10,3

A total of 41.22% of the nurses had previously undergone aesthetic surgery or aesthetic procedures. The percentage of patients whose relatives had undergone aesthetic surgery or procedures was 54.45%. A total of 68.39% of the nurses thought of having undergone aesthetic surgery or a procedure when there was an area they did not like on their body. The areas most desirable for aesthetic procedures were the face, forehead, and cheek areas (28.18%) (Table 3).

**Table 3:** Aesthetic surgery attitudes of participants

Variables	*N*	%
**Have you had any aesthetic surgery or procedure before?**		
Yes	413	41,2
No	519	58,8
**Have any of your relatives ever had an aesthetic surgery or procedure?**		
Yes	547	54,4
No	457	45,6
**Do you have plastic surgery or procedures when you don’t like any part of your body?**		
Yes	686	68,4
No	318	31,6
The area where you want to have an aesthetic procedure * ( *n* ** = 1732)**		
Nose	194	11,2
Meme	218	12,5
Face-Beach-Dish	489	28,2
Lip	378	21,8
Hip	89	5,1
Abdomen	247	14,2
Neck	43	2,5
Eyelid	65	3,8
Ear	9	0,7

* More than one answer was given

The average scores for the desire to be liked were $\overline X$=22.99, social appearance anxiety was $\overline X$=53.77, and aesthetic surgery acceptance was $\overline X$=57.61 (Table 4).

**Table 4:** Scale averages, correlation analysis results

Sclaes and subscales	Stat.	Min-Max.	$\overline X$	SS	1	2	3
1. Desire to be liked	9	9–36	22,99	6,61	1	0,710*	0,619*
2. Social appearance anxiety	16	16–80	53,97	13,48		1	0,717*
3. Aesthetic Surgery acceptance	15	1-105	57,61	20,07			1

* *p* < 0,01

When the correlation analysis results were examined, a statistically significant positive and strong relationship was found between the desire to be liked and social appearance anxiety and acceptance of aesthetic surgery (*r* > 0.500, *p* < 0.01) (Table 4).

The multiple regression model to determine the acceptance of aesthetic surgery and its determinants was statistically significant (F (2,1001) = 26.368, *p* = 0.000). According to the analysis results, the independent variables (desire to be liked and social appearance anxiety) explained 31.8% of the change in the dependent variable (aesthetic surgery acceptance). These results showed that aesthetic surgical acceptance is affected by the desire to be liked and by social appearance anxiety (Table 5).

**Table 5:** Aesthetic surgery Acceptance and determinants, multiple regression analysis

Değişkenler	B	SH	β	t	*p*
Constant	25,625	2,161		11,860	0,000
Desire to be liked	1,004	0,129	0,331	7,774	0,000
Social appearance anxiety	1,053	0,435	0,171	3,801	0,000
**Adjusted R²=0.318**	**F = 26.368**			*p* < 0.01	
**Dependent Variable**: Aesthetic Surgery acceptance					

## Discussion

In this study, it was determined that there was a strong positive relationship between the desire to be liked, social appearance anxiety and aesthetic surgery acceptance in nurses. As a result of the literature review, the present study is the first study conducted on nurses. In this respect, the study adds originality to the literature. For this reason, the findings of the study were discussed in line with the literature, with similar studies as much as possible.

In the present study, all of the nurses used social media; the most commonly used social media account was Instagram, and they spent an average of 2–3 h a day on social media. The social media platform with the highest number of users worldwide is Facebook, and Instagram ranks fourth. A total of 30.6% of Instagram users were aged 25–34 years. India has the highest number of Instagram users worldwide, with 362 million users, and Türkiye ranks fourth, with 57 million users [48]. In a study conducted by Kerr et al. (2020), it was determined that nurses shared posts on their Instagram accounts to promote and improve health, and even these nurses became a phenomenon [49]. In a study examining the social media accounts of student nurses in China, it was determined that student nurses shared posts that did not comply with professional ethics [50]. In the study conducted by Karadaş et al. (2021) with nursing students, it was determined that students were affected by the negative posts of the people they follow. Similarly, studies in the literature have shown that nursing students adopt an image of nonprofessional nursing on social media [50–52]. These results indicate that nurses should stay away from posts that violate professional ethics and patient privacy while using social media platforms. In addition, by providing awareness training to nurses on social media communication, both the protection of professional ethics and patient privacy violations can be prevented. In addition, guidelines should be prepared, especially for nurses, for social media communication.

As a result of the study, 41.2% of the nurses had previously undergone an aesthetic procedure, 68.4% of them preferred an aesthetic procedure when there was an area they did not like in their bodies, and 28.2% of them wanted to have an aesthetic procedure on the face, forehead or cheek area. In a study conducted by Alcan and Çetin (2022) on women’s acceptance of aesthetic surgery, it was determined that 10.2% of married women had aesthetic procedures, 63.5% wanted to have aesthetic procedures in areas they did not like in their body, and 21% wanted to have breast aesthetics [53]. According to the data of the International Society of Aesthetic Plastic Surgery (ISAPS) 2022, the most common aesthetic procedures performed in the face-cheek-forehead area are eyelid surgery (Eyelid Surgery), rhinoplasty (Rhinoplasty), lip augmentation (Lip Enhancement), and cheek filling (Fat Grafting - Face) [3]. According to these results, individuals generally prefer to perform aesthetic procedures on more visible areas.

A strong positive relationship was found between aesthetic surgery acceptance and desire to be liked and between aesthetic surgery acceptance and social appearance anxiety (*r* > 0.500, *p* < 0.01). In the present study, the mean score on the aesthetic surgery acceptance scale was $\overline X$=57.61, the mean score on the social appearance anxiety scale was $\overline X$=53.77, and the mean score on the desire to be liked scale was 22.99 (*p* < 0.01). According to the results of the regression analysis, aesthetic surgery acceptance is affected by the desire to be liked and by social appearance anxiety. In the study conducted by Bakşi and Tuncer (2021) with nursing students, the mean of the aesthetic surgery acceptance scale $\overline X$=56.11 was determined. In the study conducted by İşeri and Şen Atasayar (2022) with nursing students, the aesthetic surgery acceptance scale $\overline X$=52.44 was used. In a study conducted by Göbel et al. (2023) with 1344 women, the mean social appearance anxiety was determined to be $\overline X$=36.9 [54]. In a study by Albayrak et al. (2024) involving patients who underwent rhinoplasty surgery, the mean desires to be liked before surgery were $\overline X$=19.43 and $\overline X$=12.15 after surgery [31]. In the study by Önalan et al. (2021), a strong positive relationship was found between the acceptance of aesthetic surgery and social appearance anxiety [11]. In the study conducted by Özer and Güzel (2023), a moderate positive relationship was found between social appearance anxiety and the perception of having aesthetic procedures [55]. According to these results, the desire to be liked and social appearance anxiety are important factors that direct individuals toward plastic surgery. Body dissatisfaction is a result of negative thoughts about one’s own body. These individuals may turn to plastic surgery due to psychosocial factors, social media and peer influence. Moreover, body dissatisfaction acts as a bridge between women’s body perceptions and changing their bodies. In addition, the discourse that having a beautiful physical appearance is a requirement of being feminine supports this situation and causes individuals to turn to plastic surgery [56, 57]. According to the studies conducted, negative behaviors such as not finding oneself attractive, the desire to be liked, being uncomfortable with one’s physical appearance and feeling shame about one’s body are among the factors that positively affect individuals with social appearance anxiety toward plastic surgery. Individuals experiencing such anxiety try to reduce the risk of social exclusion and reduce the level of anxiety by creating a positive situation in their self-evaluation status through aesthetic procedures [11, 16, 31, 49]. The results obtained and the literature show that the desire to be liked and social appearance anxiety are among the most important factors influencing aesthetic surgery acceptance among nurses, as is the case for all women. Social appearance anxiety and the desire to be liked are related to negative body satisfaction [40]. Nurses, who are responsible for the health and care of patients, may experience many negative effects of body dissatisfaction. First, body dissatisfaction can lead to serious decreases in nurses’ self-confidence. This may cause nurses to feel inadequate at work and in their social environment. In particular, body image concerns may negatively affect nurses’ professional performance and increase the likelihood of distraction and error making. Moreover, nurses who experience body dissatisfaction may have high levels of stress and anxiety, which may increase the risk of burnout syndrome at work [58, 59]. Body dissatisfaction can lead to social isolation and depression, reducing nurses’ overall quality of life. This can negatively affect both nurses’ personal health and the quality of patient care because nurses’ mental and physical health directly affects their job performance [60, 61]. Therefore, psychological support and professional development programs are important for minimizing the effects of body dissatisfaction on nurses.

The current study, like all studies, has several limitations. First, because it was a cross-sectional study, we were unable to determine the causal relationships between the desire to be liked, social appearance anxiety, and acceptance of plastic surgery. Second, all the data were collected using self-report questionnaires, a process that leads to inevitable reporting bias. Third, the participants were only Turkish nurses, limiting the generalizability of the findings to nurses working in other countries. Future empirical longitudinal studies with nurses working in different countries are needed to establish causal relationships and distinctions between variables.

## Practical implications

The practical implications of this study are important for improving nurses’ psychological well-being and professional performance. Understanding the effects of social appearance anxiety and the desire to be liked on nurses’ acceptance of plastic surgery highlights the need for targeted interventions in the healthcare sector. The results suggest that psychological support programs are necessary to help nurses manage social appearance anxiety and desire to be liked. As a result of these support programs, awareness can be raised among nurses about the potential psychological effects of striving for social standards of beauty. The study results emphasize the high use of social media among nurses. Institutions can create guidelines and training sessions on maintaining professional ethics online, ensuring that social media use does not contribute to negative body image or nonprofessional behavior. By implementing these practical measures, policy developers and healthcare providers can reduce the negative effects of social appearance anxiety and desire to be liked, leading to a healthier and more productive nursing workforce. Thus, nurses who have knowledge about psychosocial behaviors that lead individuals to plastic surgery, such as social appearance anxiety and the desire to be liked, can educate those who want to have unnecessary aesthetic procedures for these reasons in the community and in their immediate surroundings. As a result, in addition to preventing unnecessary aesthetic procedures, additional financial burdens are prevented, and these procedures contribute to the positive image of nurses in society. Moreover, nurses contribute to the protection and development of public health and increase the level of health education in society while setting an example for young people who will practice this profession after them.

## Conclusion

The present study revealed that nurses experienced moderate social appearance anxiety and a desire to be liked. According to the results, there were strong positive relationships between social appearance anxiety and the desire to be liked and aesthetic surgery, and aesthetic surgery acceptance was affected by the desire to be liked and social appearance anxiety. In addition, it was determined that nurses are candidates for aesthetic surgery procedures throughout their lives.

## Data Availability

The datasets used and/or analysed during the current study are available from the corresponding author on reasonable request.
